# Identification and Molecular Mechanism of Anti-inflammatory Peptides Isolated from Jack Bean Protein Hydrolysates: in vitro Studies with Human Intestinal Caco-2BBe Cells

**DOI:** 10.1007/s11130-024-01201-x

**Published:** 2024-06-28

**Authors:** Bambang Dwi Wijatniko, Yoshinari Yamamoto, Makoto Hirayama, Takuya Suzuki

**Affiliations:** 1 Graduate School of Integrated Sciences for Life, Hiroshima University, Higashi-Hiroshima, 739- 8528 Japan; 2 Department of Food and Agricultural Product Technology, Universitas Gadjah Mada, Yogyakarta, 55281 Indonesia

**Keywords:** Anti-inflammatory Peptides, Jack bean, Isolation, Intestinal Cells, Interleukin-8, Tumor Necrosis factor-α

## Abstract

Jack bean (JB), *Canavalia ensiformis* (L.) DC, is a commonly cultivated legume in Indonesia. It is rich in protein, which can be hydrolyzed, making it potentially a good source of bioactive peptides. Intestinal inflammation is associated with several diseases, and the production of interleukin-8 (IL-8) in intestinal epithelial cells induced by tumor necrosis factor (TNF)-α has an important role in inflammatory reaction. The present study investigated the anti-inflammatory effects of peptides generated from enzymatic hydrolysis of JB protein on human intestinal Caco-2BBe cells. Additionally, in silico approaches were used to identify potential bioactive peptides. JB protein hydrolysate (JBPH) prepared using pepsin and pancreatin reduced the IL-8 expression at protein and mRNA levels in Caco-2BBe cells stimulated with TNF-α. Immunoblot analysis showed that the JBPH reduced the TNF-α-induced phosphorylation of c-Jun-NH(2)-terminal kinase, nuclear factor kappa B (NF-κB), and p38 proteins. Anti-inflammatory activity was observed in the 30% acetonitrile fraction of JBPH separated on a Sep-Pak C18 column. An ultrafiltration method revealed that relatively small peptides (< 3 kDa) had a potent inhibitory effect on the IL-8 production. Purification of the peptides by reversed-phase and anion-exchange high performance chromatography produced three peptide fractions with anti-inflammatory activities. A combination of mass spectrometry analysis and in silico approaches identified the potential anti-inflammatory peptides. Peptides derived from JB protein reduces the TNF-α-induced inflammatory response in Caco-2BBe cells via NF-κB and mitogen-activated protein kinase signaling pathways. Our results may lead to a novel therapeutic approach to promote intestinal health.

**Supplementary Information:**

The online version contains supplementary material available at 10.1007/s11130-024-01201-x.

## Introduction

Jack bean (JB, *Canavalia ensiformis* (L.) DC) is a protein-rich legume commonly cultivated in Indonesia. It is highly productive at harvest and can be grown on marginal land with low nutrients. JB can be a good source of essential amino acids [1] and a promising protein alternative to soybeans, which are widely utilized worldwide. Despite its nutritional advantage, the presence of anti-nutritional substances such as tannin, hydrogen cyanide, and phytate limits the consumption of JB [2, 3]. Jack bean protein is mainly composed by Canavalin as major storage protein of the jack bean and belongs to the classical vicilin fraction. Additionally, Concanavalin A and B is renowned as lectin presented in jack bean [4]. A growing body of evidence suggested jack bean protein showed beneficial physiological function, including anti-oxidative and anti-cholesterol [4, 5]. Another study suggested that fermented jack bean (jack bean tempeh) exhibited angiostensin-converting enzyme (ACE) inhibitory effect [6]. JB protein is rich in hydrophobic amino acids such as leucine, valine, proline, and alanine [7]. Previous studies show that most bioactive peptides that exert beneficial physiological effects on human health are rich in the hydrophobic amino acids. These peptides penetrate into the intestinal mucosa and interact with target molecules to show biological effect [8]. Indeed, it has been reported that protein hydrolysates derived from JB protein exhibit various biological activities such as radical scavenging activity, ferric reducing power, and angiotensin-converting enzyme inhibitory activity [9, 10]. A variety of proteins in jack bean provide a material basis for diverse peptides after digestion. Thus, the JB protein can be a promising source of bioactive peptides. Accumulating evidence demonstrates that intestinal inflammation is involved in both intestinal and non-intestinal diseases [11]. Although intestinal inflammation is induced by a wide range of stimuli, tumor necrosis factor (TNF)-α expressed by activated immune cells such as macrophages and dendritic cells upregulates the production of other pro-inflammatory cytokines in various cells [12]. Interleukin-8 (IL-8) is produced in intestinal epithelial cells in response to TNF-α and plays a critical role in recruiting neutrophils during inflammatory conditions [13]. The excessive neutrophil activation contributes to tissue damage and the pathogenesis of inflammatory diseases. Accordingly, the suppression of IL-8 production by dietary peptides may be an effective approach to promote intestinal health [14, 15].

The present study aimed to produce anti-inflammatory peptides from JB protein by enzymatic digestion using pepsin and pancreatin. To investigate the anti-inflammatory effect of peptides from jack bean protein hydrolysate (JBPH) and their underlying mechanisms, a well-known human intestinal cell model, Caco-2Bbe, stimulated with TNF-α was used in the study. In addition, for the identification of potential peptides responsible for the anti-inflammatory activity, an in silico approach was used.

## Materials and methods

The material and methods section is presented as supplementary material.

## Results and Discussion

### Effect of JBPH on Viability of Caco-2BBe Cells

**Fig. 1:**
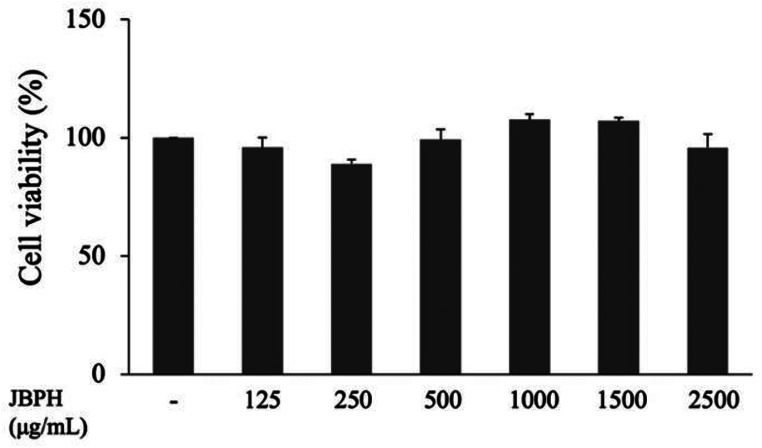
Effect of JBPH on viability of Caco-2BBe cells. Cells were incubated with JBPH for 24 h and the cell viability was evaluated using a commercially available kit. Values are presented as the mean ± standard error of mean (*n* = 5)

Because dietary peptides should be non-toxic to the intestinal cells, the effect of JBPH on the viability of Caco-2BBe cells was investigated. The treatment of cells with JBPH at concentrations up to 2500 µg/mL for 24 h did not influence the viability of Caco-2BBe cells (Fig. 1).

### Anti-inflammatory Effect of JBPH

**Fig. 2:**
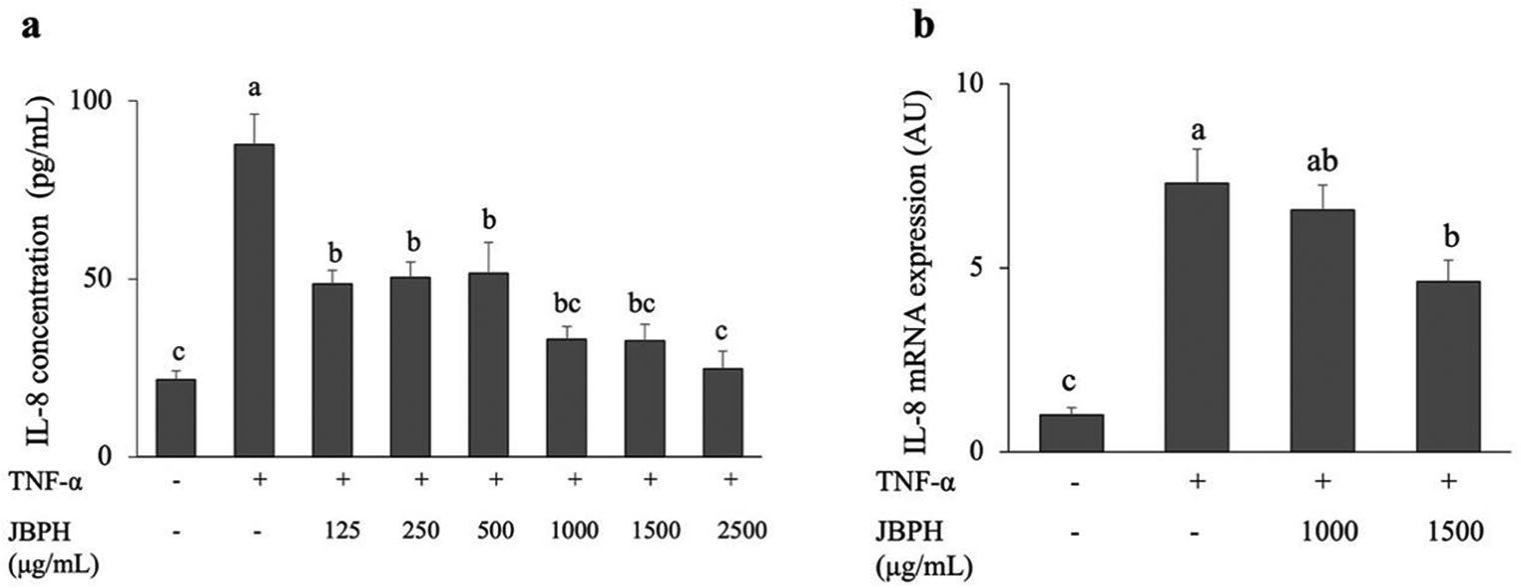
Effect of JBPH on IL-8 protein and mRNA expression in Caco-2BBe cells. Caco-2BBe cells were incubated with and without JBPH in the presence and absence of TNF-⍺. The IL-8 protein production was determined by ELISA (**a**). The IL-8 mRNA expression was determined by qRT-PCR analysis (**b**). Values are presented as the mean ± SEM (*n* = 5). Means not marked by a common letter are significantly different as determined using the Tukey–Kramer’s *post-hoc* test (*P* < 0.05)

The production of IL-8 in intestinal epithelial cells induces the neutrophil recruitment to inflammatory site and is closely involved in the pathogenesis of various intestinal diseases [16]. Figure 2a shows that TNF-α treatment substantially increased IL-8 production in intestinal Caco-2BBe cells compared to that in control cells, indicating an inflammatory response. Pre-treatment of cells with JBPH reduced the TNF-α-induced IL-8 production in a more or less dose-dependent manner. The results showed that JBPH at 1000, 1500 and 2500 µg/mL potently suppressed the IL-8 production, indicated by the observation that the IL-8 levels with these treatments were not significantly different from the control cells. Therefore, the cells incubated with 1000 and 1500 µg/mL JBPH were subjected to qRT-PCR analysis of the *IL-8* mRNA expression. Consistent with the protein levels of IL-8, JBPH reduced the *IL-8* mRNA expression in a dose-dependent manner in the TNF-α-treated Caco-2BBe cells (Fig. 2b).

### Cellular Mechanism of Anti-inflammatory Effect of JBPH

**Fig. 3:**
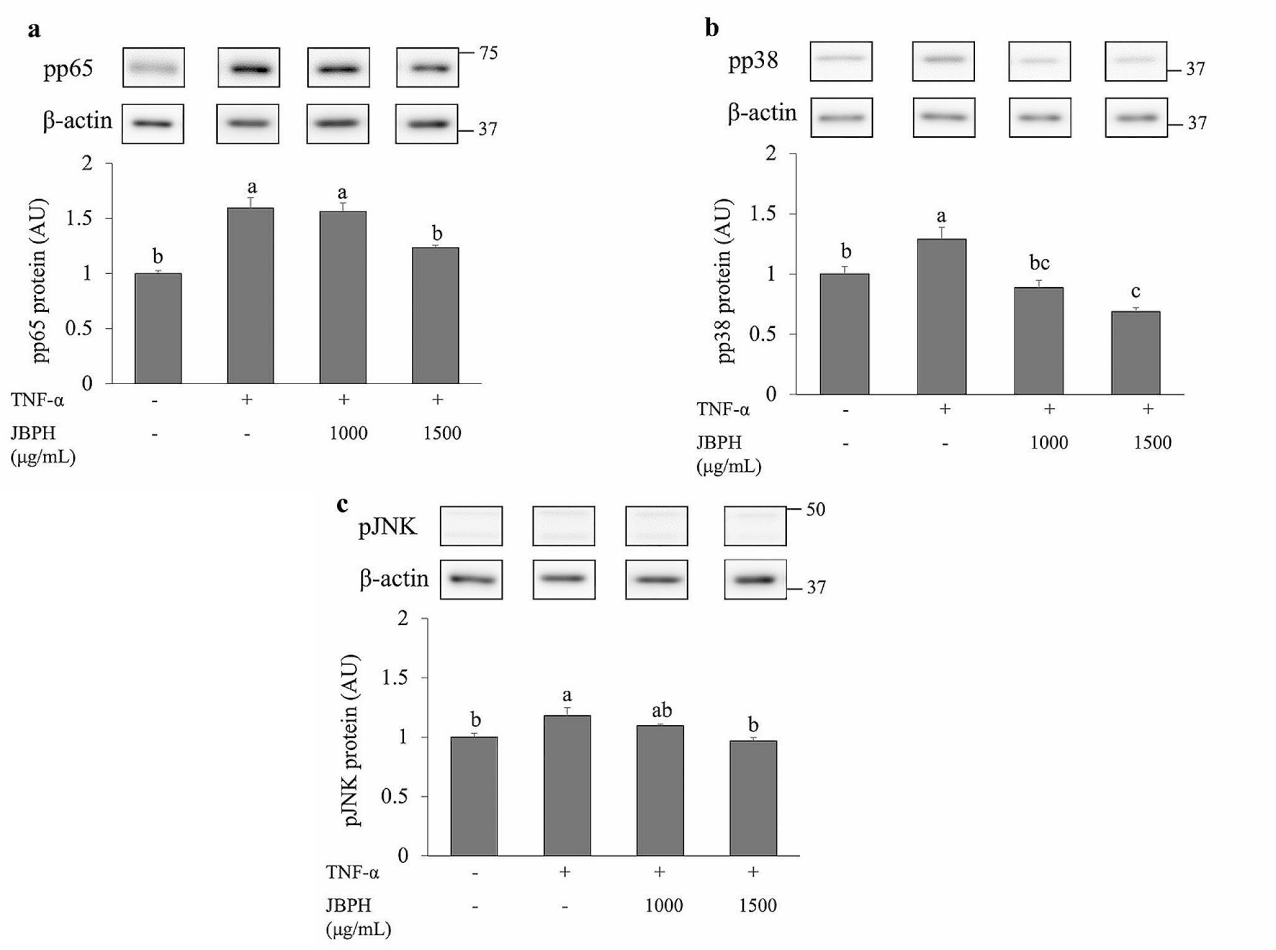
Effect of JBPH on phosphorylation of NF-κB and MAPK signaling molecules. Caco-2BBe cells were incubated with and without JBPH in the presence and absence of TNF-α. Phosphorylation of p65 (**a**), p38 (**b**), and JNK (**c**) was determined by immunoblot analysis. Values are presented as the mean ± SEM (*n* = 5). Means not marked by a common letter are significantly different as determined using the Tukey–Kramer’s post-hoc test (*P* < 0.05)

It has been reported that TNF-α transcriptionally upregulates the IL-8 expression through various signaling pathways such as NF-κB and MAPK [17]. Immunoblot analysis (Fig. 3a-c) showed that the stimulation of Caco-2BBe cells with TNF-α upregulated the phosphorylation of p65, p38, and JNK, indicating cellular activations. Pre-treatment with JBPH suppressed the phosphorylation of p65, p38 and JNK proteins caused by TNF-α stimulation in a dose-dependent manner. These results suggest that JBPH suppresses inflammatory signaling induced by TNF-α to decrease the IL-8 transcription. The regulation of NF-κB, p38,and JNK pathways seems to be a central event in the JBPH-mediated suppression of IL-8 production.

TNF-α is known to activate these cellular signaling pathways through TNF receptors, resulting in the IL-8 expression in intestinal epithelial cells. Upon NF-κB activation and translocation into the nucleus, it binds to the promoter region of the *IL-8* gene, thereby initiating the transcription of *IL-8* mRNA. JNK and p38 also activate the downstream transcription factors such as AP-1, c-Jun, ATF-2, and CREB, which induce the transcription of IL-8 [18, 19]. Previous studies demonstrated that peptides derived from egg-white and a dipeptide derived from ovotransferrin effectively suppressed the activation of NF-κB and MAPK pathways [20, 21]. Accordingly, our results suggest that the reduced activation (phosphorylation) of NF-κB, p38, and JNK by JBPH results in the downregulation of IL-8 transcription in the cells.

### Anti-inflammatory Effect of Peptides Fractionated from JBPH

Fractionation of JBPH was performed in a sequential step to isolate potent anti-inflammatory peptides. First, JBPH was subjected to a Sep-Pak C18 cartridge using a three-step acetonitrile gradient elution (15, 30, and 45%). Based on the BCA assay, the recovery ratio of the peptide in the 15, 30, and 45% acetonitrile fractions were 17.9, 52.7, and 15.3%, respectively. However, when JBPH was eluted at higher acetonitrile concentration (65%), the recovery ratio of peptide was very low (1.44%). Therefore, the three fractions from the lower acetonitrile concentrations were administered to Caco-2BBe cells at concentration of 1000 µg/mL 6 h before TNF-α stimulation.

**Fig. 4:**
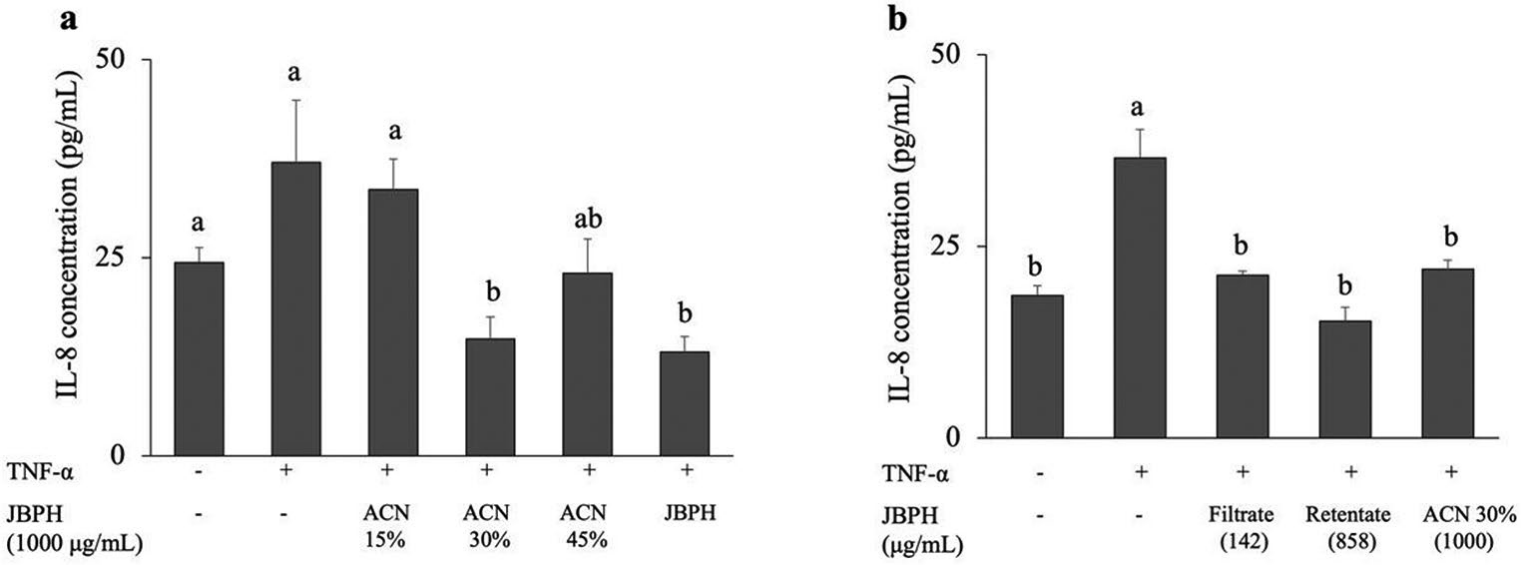
Effect of JBPH on IL-8 protein levels in Caco-2BBe cells. Caco-2BBe cells were incubated with and without different JBPH in the presence and absence of TNF-α. The IL-8 protein production was determined by ELISA. The three fractions of JBPH were prepared by a step-gradient separation on Sep-Pak C18 cartridges (**a**); and JBPH were separated by ultrafiltration with 3 kDa molecular weight cut-off (**b**). Values are presented as the mean ± SEM (*n* = 5). Means not marked by a common letter are significantly different as determined using the Tukey–Kramer’s *post-hoc* test (*P* < 0.05)

Among the three fractions, suppression of IL-8 production by the 30% acetonitrile fraction was roughly comparable to that of the original JBPH (Fig. 4a). Next, the 30% acetonitrile fraction was further separated using ultrafiltration with 3 kDa MWCO. Based on the BCA assay, 14 and 86% peptides from the 30% acetonitrile fraction were distributed in the filtrate (< 3 kDa) and retentate (> 3 kDa) fractions, respectively.

Interestingly, both fractions suppressed the IL-8 production in the TNF-α-stimulated Caco-2 BBe cells in a similar manner (Fig. 4b); peptides in the filtrate fraction with a relatively low protein concentration (142 µg/mL) exhibited IL-8 suppression similar to those by the retentate fraction (858 µg/mL) and the original sample. This suggested that the peptides in the filtrate fraction also had potent anti-inflammatory activity.

**Fig. 5:**
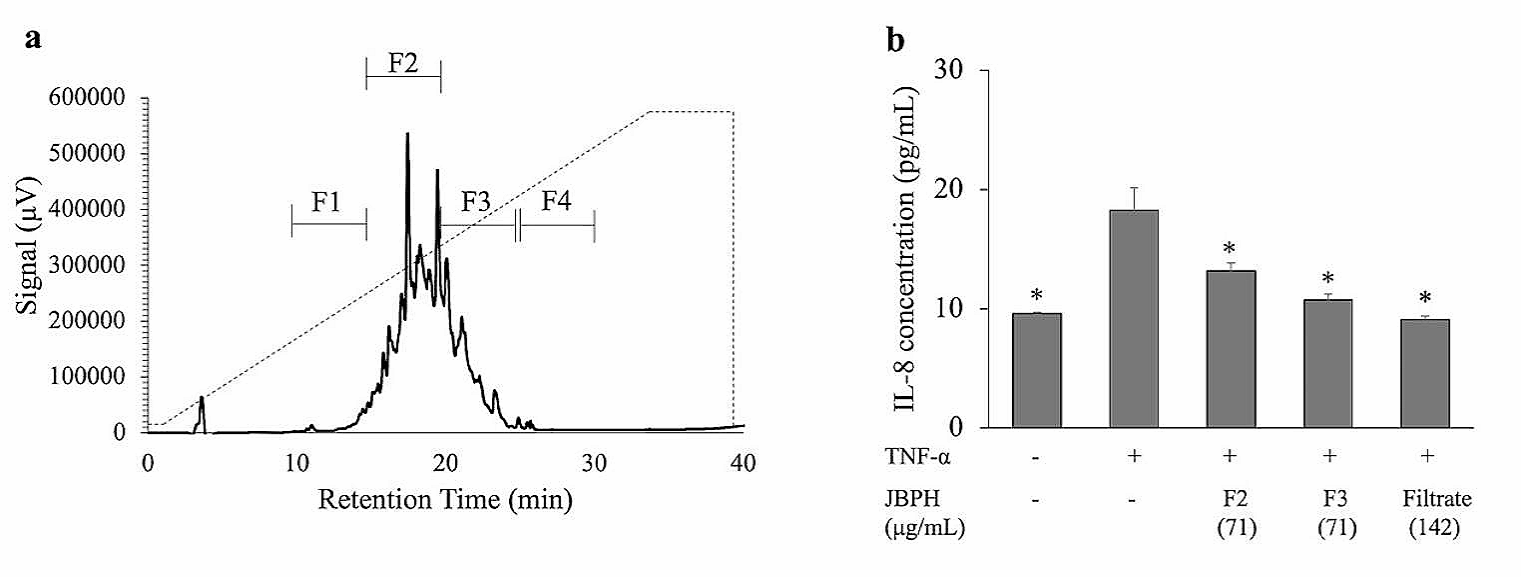
Separation of JBPH with RP-HPLC and its effect on IL-8 protein in Caco-2BBe cells. The chromatogram of JBPH by RP-HPLC was shown. The dashed line indicated the acetonitrile gradient for elution (**a**). Caco-2BBe cells were incubated with and without jack bean protein hydrolysates (JBPH) RP-HPLC fractions in the presence and absence of TNF-α. The IL-8 protein production was determined by ELISA (**b**). Values are presented as the mean ± SEM (*n* = 5). *P < 0.05 compared to the control (+ TNF-⍺) as determined using Dunnet’s post-hoc test

Accordingly, the filtrate fraction was further separated using RP-HPLC. As shown in Fig. 5a, four fractions (F1–F4) were collected based on the retention time, of which anti-inflammatory activity was tested in fractions F2 and F3 in Caco-2BBe cells since the peptides were mostly distributed in these two fractions according to the BCA assay. Both the fractions suppressed the TNF-α-induced IL-8 production (Fig. 5b).

**Fig. 6:**
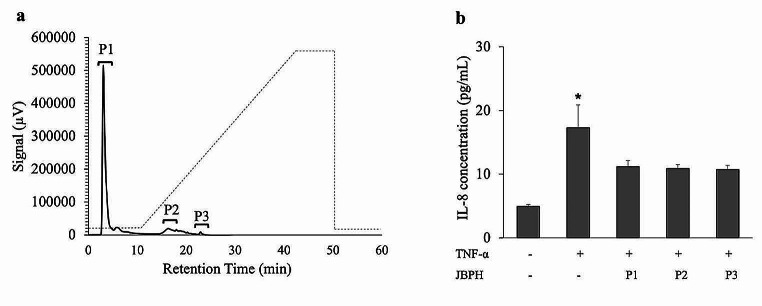
Separation of JBPH with anion exchange HPLC and its effect on IL-8 protein in Caco-2BBe cells. The chromatogram of JBPH by anion exchange HPLC was shown. The dashed line indicated the NaCl gradient for elution (**a**). Caco-2Bbe cells were incubated with and without jack bean protein hydrolysates (JBPH) anion exchange HPLC fractions in the presence and absence of TNF-α. The IL-8 protein production was determined by ELISA (**b**). Values are presented as the mean ± SEM (*n* = 5). *P < 0.05 compared to the negative control (without TNF-⍺) as determined using Dunnet’s post-hoc test

The result also revealed that the suppression by F3 was comparable to the original filtrate fraction. Furthermore, the peptides in F3 may be more hydrophobic than those in F2, as they were eluted later in the RP-HPLC. Considering that hydrophobicity is an important feature in the biological activity of peptides [22, 23], F3 was subjected to separation using anion exchange chromatography. As shown in Fig. 6a, three peaks designated as P1, P2, and P3 were collected, and the anti-inflammatory activities and cytotoxicity were examined in Caco-2BBe cells. All three peaks (P1, P2, and P3) suppressed the IL-8 production induced by TNF-α without any cytotoxicity (Fig. 6b and Fig. S1). Our results also suggest that different hydrophobic peptides with relatively small molecular weights (< 3 kDa) possibly have a role in the regulation of inflammatory response in Caco-2BBe cells. We found that the anti-inflammatory peptides isolated from JBPH are of low molecular weight (< 3 kDa) with hydrophobic nature.

Screening of bioactive peptides in previous studies suggested that peptide with molecular weight below 3 kDa exhibited greater bioactivities such as anti-inflammatory, anti-oxidative, and anti-microbial activities compared to larger peptides [24–26]. This is not unexpected, since small peptides may be easily taken up by cells and thus exhibit bioactivity. The C18 reverse phase column enables the separation of peptides based on the hydrophobic interaction of peptides between the elution buffer and the stationary phase [27]. Peptides isolated from JBPH are likely to have high hydrophobicity because they eluted at acetonitrile concentrations between 68 and 82% and were collected as the F3 fraction. Similar to our study, it has been reported that plant-derived bioactive peptides with high hydrophobic properties effectively reduce the inflammatory response by inducing membrane depolarization and disrupting the inflammatory cascade pathway due to their high affinity to cell membranes in jejunal epithelial cell line [8].

### Identification of Potential Anti-inflammatory Peptides

To identify the amino acid sequences of peptides, the fractions P1, P2, and P3 were subjected to the *de novo* peptide sequencing with mass spectrometry, which identified 267, 72, and 64 peptides in fractions P1, P2, and P3, respectively. Using a cutoff average local confidence (ALC) of ≥ 85% on a *de novo* peptide sequencing that represented the spectrum quality, we obtained 77, 31, and 19 peptides from P1, P2, and P3, respectively (Table S2, S3, and S4). In National Center for Biotechnology Information (NCBI) database, only five proteins, Canavalin, Concanavalin-A, Concanavalin-B, Alpha-mannosidase, and Urease, were found as parent proteins of these peptides. Using the in silico approach with PeptideRanker score, BIOPEP peptide database, and PreTP-EL, 23 peptides were identified as the anti-inflammatory peptides (Table 1). Specifically, the BIOPEP, PreTP-EL, and PeptideRanker identified 6, 8, and 9 peptides with amino acid lengths ranging from 4 to 13. BIOPEP is a database of peptides that show biological activity and has become a standard tool for screening and identifying the potential bioactive peptides [28]. BIOPEP can also predict the potential bioactivity of the parent peptides and their cleavage. In this study, BIOPEP has identified three peptides in JBPH, DDYFPYADGRNA, YWGQREDGL, and ANPRHLDAGGKA, which possibly release the anti-inflammatory peptides, PY, YW, and ANP, respectively. We also determined that the parent proteins of DDYFPYADGRNA and YWGQREDGL are α-mannosidase and Concanavalin B, respectively. PreTP-EL is a novel therapeutic peptide prediction method using ensemble learning that combines individual predictors through a genetic algorithm. It provides information on whether the peptide sequences entered into the query are anti-inflammatory peptides or not [29]. In this study, PreTP-EL has identified eight peptides as potential anti-inflammatory peptides, including LLNPDNNQNLRLL with the highest score (Table S5). Although Canavalin has been found to be the parent protein of the FLSSTKRLPSYL, other seven peptides are still orphan. PeptideRanker is a bioactive peptide prediction server based on a novel N-to-1 neural network and used in many bioactive peptide studies [30, 31]. It predicted three peptides with high scores from the P1, P2, and P3 fractions, indicating that they may have anti-inflammatory properties. The peptides include WTGF, FGLM, RLFG, FVDF, FDYL, HFDY, DLDF, FEEF and YEGLDF.

**Table 1:** Potential anti-inflammatory peptides in jack bean protein hydrolysates

Fraction	Parent protein	Peptide	m/z	RT	Area	ALC (%)	Error (ppm)	PeptideRanker score	Potential anti-inflammatory activity
P1	NF	NLANPSRADF	552.7759	15.39	5.25E + 07	96	1.2	0.3720	Contain sequence of ANP^a^
	NF	PYLPDL	717.3842	18.86	2.17E + 07	93	3.5	0.7503	Contain sequence of PY^a^
	NF	LEVNPYL	847.4583	18.05	3.35E + 06	95	2.7	0.2315	Contain sequence of PY^a^
	NF	LRKPEL	378.2428	13.8	6.11E + 07	96	1.2	0.1941	AIP^b^
	NF	PLLLGAHE	424.7535	15.16	5.02E + 07	95	1.1	0.2078	AIP^b^
	NF	NTPVRKLEKL	599.3696	13.85	4.08E + 07	91	0.5	0.1606	AIP^b^
	NF	LLLPH	296.6952	15.83	3.48E + 07	93	2.3	0.4103	AIP^b^
	Canavalin	FLSSTKRLPSYL	706.4004	16.45	2.44E + 07	98	-0.6	0.4510	AIP^b^
	NF	FRKHLLA	884.5458	13.38	7.83E + 06	89	-0.8	0.4469	AIP^b^
	NF	LLNPDNNQNLRLL	768.9291	17.25	7.53E + 06	86	1.1	0.4717	AIP^b^
	NF	LELL	487.3145	18	7.01E + 06	92	3.8	0.1783	AIP^b^
	NF	WTGF	510.236	18	7.38E + 06	95	2.6	0.9695	Potential bioactive peptides^c^
	NF	FGLM	467.2322	18.38	1.30E + 07	86	0	0.9673	Potential bioactive peptides^c^
	NF	RLFG	492.2942	14.6	2.65E + 07	85	2.7	0.8709	Potential bioactive peptides^c^
P2	Chain B, α-mannosidase	DDYFPYADGRNA	702.297	16.81	1.72E + 06	87	0.4	0.4921	Contain sequence of PY^a^
	Chain A, Concanavalin B	YWGQREDGL	562.2622	16.46	1.21E + 06	92	0.5	0.4033	Contain sequence of YW^a^
	NF	FVDF	527.2499	18.11	6.63E + 06	96	-0.1	0.8720	Potential bioactive peptides^c^
	NF	FDYL	557.2605	17.99	2.83E + 06	94	-0.1	0.8516	Potential bioactive peptides^c^
	NF	HFDY	581.2361	14.88	1.03E + 07	96	1.1	0.6857	Potential bioactive peptides^c^
P3	NF	ANPRHLDAGGKA	402.8829	12.02	1.31E + 07	94	0.4	0.4180	Contain sequence of ANP^a^
	NF	DLDF	509.2238	18.44	2.06E + 05	89	-0.7	0.7161	Potential bioactive peptides^c^
	NF	FEEF	571.2404	17.25	7.34E + 06	98	0.9	0.5781	Potential bioactive peptides^c^
	NF	YEGLDF	743.3251	18.48	7.88E + 05	88	0.7	0.5015	Potential bioactive peptides^c^

NF, not found; RT, retention time; ALC, average local confidence; AIP, anti-inflammatory peptide.

^a^from BIOPEP (https://biochemia.uwm.edu.pl/biopep/start_biopep.php).

^b^ from PreTP-EL (http://bliulab.net/PreTP-EL).

^c^ from PeptideRanker (http://distilldeep.ucd.ie/PeptideRanker/).

However, the anti-inflammatory activity of JBPH was not examined in in vivo using animal models in the present study. Murine models of experimental colitis induced by dextran sulfate sodium and 2,4,6-trinitrobenzene sulfonic acid have been used to study the anti-inflammatory activities of dietary factors [32]. It is important for the bioactive peptides to be resistant to digestive enzymes such as pepsin, trypsin, and chymotrypsin. It is very likely that the JBPH is resistant to the digestive enzyme and reaches the site of inflammation in intact forms in the intestine, because was prepared by hydrolysis using pepsin and pancreatin. The anti-inflammatory activity of JBPH using in vivo models will be investigated in future studies.

## Conclusion

Hydrolysis of JB protein using pepsin and pancreatin generates potential anti-inflammatory peptides. The peptides reduce the IL-8 expression by suppressing the NF-κB and MAPK signaling pathways in the TNF-α-stimulated Caco-2BBe cells. In addition, the in silico approaches revealed the sequences of potential anti-inflammatory peptides. Taken together, our findings may contribute to the development of nutraceutical, especially from underutilized plant-based protein hydrolysates, to promote intestinal health. However, further studies are needed to investigate the biological functions of these peptides in in vitro and in vivo studies.

## Electronic Supplementary Material

Below is the link to the electronic supplementary material.

Supplementary Material 1

## Data Availability

No datasets were generated or analysed during the current study.

## Abbreviations

AU: Arbitrary unit
ELISA: Enzyme-linked immunosorbent assay
IL-8: Interleukin-8
JBPH: Jack bean protein hydrolysates
JNK: c-Jun-NH(2)-terminal kinase
MAPK: Mitogen-activated protein kinase
MS: Mass spectrometry
NF-κB: Nuclear factor kappa B
qRT-PCR: Real-time reversed transcription polymerase chain reaction
RP-HPLC: Reversed-Phase High-Performance Liquid Chromatography
SEM: Standard error of mean
TNF-α: Tumor necrosis factor-α
