# Supplementary material for: Identification and Molecular Mechanism of Anti-inflammatory Peptides Isolated from Jack Bean Protein Hydrolysates: in vitro Studies with Human Intestinal Caco-2BBe Cells
Source: Plant Foods Hum Nutr. 2024 Jun 28;79(3):624–31. doi: 10.1007/s11130-024-01201-x (PMC11410891; doi:10.1007/s11130-024-01201-x)
Supplement: Supplementary file 1 — Supplementary Material 1 [file 11130_2024_1201_MOESM1_ESM.docx]

**Supplementary materials**

**Identification and molecular mechanism of anti-Inflammatory peptides isolated from jack bean protein hydrolysates: *in vitro* studies with human intestinal Caco-2BBe cells**

**Bambang Dwi Wijatniko^ab^, Yoshinari Yamamoto^a^, Makoto Hirayama^a^,** **Takuya Suzuki^a*^**

^a^ Graduate School of Integrated Sciences for Life, Hiroshima University, Higashi-Hiroshima, 739-8528, Japan

^b^Department of Food and Agricultural Product Technology, Universitas Gadjah Mada, Yogyakarta, 55281, Indonesia

^*^**Correspondence to**:

Takuya Suzuki,

Graduate School of Integrated Sciences for Life, Hiroshima University,

Higashi-Hiroshima 739-8528, Japan.

E-mail : [takuya@hiroshima-u.ac.jp](mailto:takuya@hiroshima-u.ac.jp)

Tel : +81-82-424-7984

Fax : +81-82-424-7916

ORCiD: orcid.org/0000-0003-3709-543X

**Materials and methods**

**Chemicals**

JB seeds were obtained from a local farmer in Wonogiri, Central Java, Indonesia. The seeds were ground and sieved through a 250 μm mesh sieve to prepare the JB powder. Pepsin (lyophilized powder, ≥2,500 units/mg protein) was obtained from Sigma-Aldrich (St. Louis, MO, USA). Pancreatin and trifluoroacetic acid (TFA) were purchased from FUJIFILM Wako Pure Chemical (Osaka, Japan). Fetal bovine serum (FBS) and trypsin-EDTA solution were obtained from Thermo Fisher Scientific (Waltham, MA, USA). Penicillin−streptomycin (10,000 U/mL) was obtained from Nacalai Tesque (Kyoto, Japan). Cell counting kit-8 (CCK-8) was obtained from Dojindo (Kumamoto, Japan). Human TNF-α was obtained from Miltenyi Biotec (Teterow, Germany). Rabbit-phospho-stress-activated protein kinase (SAPK)/c-Jun-NH(2)-terminal kinase (JNK) (Thr183/Tyr185), rabbit-phospho-p38 mitogen-activated protein kinase (MAPK) (Thr180/Tyr182), and rabbit-phospho-nuclear factor kappa B (NF-κB) p65 (Ser536) antibodies were obtained from Cell Signaling Technology (Danvers, MA, USA). Horseradish peroxidase (HRP)-conjugated anti-rabbit and anti-mouse IgG antibodies were obtained from SeraCare Life Sciences Inc. (Milford, MA, USA). Mouse anti-β-actin antibody was purchased from Sigma-Aldrich (St. Louis, MO, USA). Acetonitrile was purchased from Merck Millipore (Burlington, MA, USA). Enzyme-linked immunosorbent assay (ELISA) kit to determine IL-8 was obtained from R&D Systems (Minneapolis, MN, USA). All other chemicals were purchased from FUJIFILM Wako Pure Chemical and Nacalai Tesque.

**JB Protein Extraction**

JB protein was isolated from JB powder as previously described [1] with slight modification. In brief, JB powder was defatted by petroleum ether (Nacalai Tesque), by dispersing it in three parts of petroleum ether (w/v), vigorously mixing with stirring for 30 min, and separating from the petroleum ether phase using a filter paper. This procedure was performed twice, followed by drying the JB powder with flowing air in a fume hood. The resulting protein powder was dissolved in four parts of ultrapure water (w/v), followed by adjusting the pH to 8.0 with 1 mol/L NaOH. The mixture was stirred for 60 min to solubilize the JB protein and centrifuged at 10,000 × *g* for 15 min (Suprema 21, Tomy Seiko, Tokyo, Japan) to obtain a protein-rich supernatant. The pH of the supernatant was adjusted to 4.5 with 1 mol/L HCl to precipitate the protein. The JB protein was collected by centrifugation (5000 × *g*, 15 min) and stored at −30 ^o^C.

**Preparation of JB Protein Hydrolysates**

The JB protein extract was dispersed in 20 parts of ultrapure water and subjected to controlled hydrolysis using pepsin and pancreatin. The protein was first hydrolyzed with pepsin at an enzyme to substrate (E/S) ratio of 1:50 (37 °C, 2 h, pH 2.0). The protein hydrolysate was heated in a boiling water bath for 10 min to inactivate the pepsin and then hydrolyzed with pancreatin at a ratio of 1:50 E/S (37 °C, 2 h, pH 7.5). The pancreatin in the mixture was heat-inactivated, similar to pepsin. The protein hydrolysate was clarified by centrifugation (2380 × *g*, 15 min), and the protein concentration in the supernatant was determined using a Bicinchoninic Acid (BCA) protein assay kit (FUJIFILM Wako Pure Chemical). The clear protein hydrolysate was desalted using Sep-Pak C18 Plus Short Cartridge (Waters, Milford, MA, USA) with 2% acetonitrile + 0.1% TFA for conditioning and washing and 65% acetonitrile + 0.1% TFA for elution. The eluate was freeze-dried (EYELA FDU-1200, New York, USA) and stored at –30 °C prior until further use.

**Step-Gradient Separation with Sep-Pak C18 cartridges**

JBPH was dissolved in 2% acetonitrile + 0.1% TFA and loaded into the Sep-Pak C18 cartridge. Peptides in the protein hydrolysates bound to the C18 matrix were eluted in a stepwise manner with 15, 30, and 45% (v/v) acetonitrile. The eluted fractions were freeze-dried and stored at −30 °C prior to the experiments.

**Ultrafiltration of the Peptide Fraction Eluted with 30% Acetonitrile**

The peptide fractions eluted with 30% acetonitrile by Sep-Pak C18 cartridge were further separated by ultrafiltration using the Amicon® Ultra-15 3K device 3 kDa (3 kDa molecular weight cut-off, Merck Millipore, Tullagreen, Carrigtwohill, Co. Cork, Ireland) centrifugal device. The freeze-dried peptides were dissolved in ultrapure water and loaded into the Amicon® Ultra-15 3K device, followed by centrifugation at 2380 × *g* for 30 min. The peptide fraction that passed through the membrane was designated as the filtrated fraction, while the fraction retained on the membrane was designated as the retentate fraction. The filtrated and retentate fractions were lyophilized and stored at –30°C prior to the experiments.

**Reversed-Phase (RP) and Anion Exchange High-Performance Liquid Chromatography (HPLC)**

The filtrate fraction obtained by ultrafiltration was further separated by reversed-phase and anion exchange HPLC (JASCO, Tokyo, Japan). The fraction was applied to the TSKgel ODS-120T column (5 µm, 4.6 mm i.d. × 250 mm, TOSOH, Tokyo, Japan) was eluted using gradient elution of water and acetonitrile with TFA 0.1% at a flow rate of 1 mL/min. The running program was set to reach gradient 0 to 100% acetonitrile in 2–35 min and maintained at 100% until 40 min. Elution was monitored at 220 nm. The peptides were collected based on the retention time. The collected fractions were freeze-dried and stored at −30 °C prior to the experiments. A specific fraction showing potent anti-inflammatory activity was subjected to further separation using an anion exchange-TSKgel DEAE-5PW column (7.5 mm i.d. × 75 mm, TOSOH) using gradient buffer of 50 mmol/L Tris-HCl (pH 7) and 1 mol/L NaCl in 50 mmol/L Tris-HCl (pH 7) at a flow rate of 1 mL/min.

**Peptide Identification by Mass spectrometry (MS)**

The peptide fractions obtained from anion exchange chromatography were subjected to the identification of amino acid sequence by Nano LC-MS/MS system equipped with the Ultimate 3000 RSLCnano system and an LTQ Orbitrap XL (Thermo Fisher Scientific). The analysis was carried out using C18 column (3 µm, 75 µm i.d. × 120 mm, Nikkyo Technos, Tokyo, Japan) with trap column (PepMap^TM^ Neo 5 μm C18 300 μm × 5 mm, Thermo Fisher Scientific) at a flow rate of 200 nL/min using mobile phase A containing 0.1% formic acid in water and mobile phase B containing acetonitrile and 0.1% formic acid. The elution program was conditioned as follows: 0–3 min, 0–4% B; 3–30 min, 4–75% B; 30–31 min, 75–90% B; 31–35 min, 90% B; 35–45 min, 4% B. Mass spectrometry was collected over 100–1000 m/z. Identification of peptide sequences was performed using de novo sequencing by PEAKS software (Bioinformatics Solutions Inc. Ontario, Canada).

**Cell culture**

Caco-2BBe cells are a clone of Caco-2 cells. It has been reported that Caco-2BBe cells morphologically and functionally comparable to those of human intestinal epithelial cells. Human intestinal Caco-2BBe cells (CRL-2102, American Type Culture Collection, Manassas, VA, USA) were maintained in Dulbecco's modified Eagle's medium (Nacalai Tesque) supplemented with 100 mL/L FBS and a penicillin-streptomycin solution under a 5% CO_2_ atmosphere at 37 °C as described previously [2]. The studies were conducted between 13 and 15 days after seeding at the passage numbers between 54 and 70. The culture medium was refreshed every 3 days.

**Cell Viability Assay**

Caco-2BBe cells were seeded in a 96-well plate (0.1 × 10^3^ cells/cm^2^) and incubated with and without JBPH (125–2500 µg/mL) and the fractions obtained by anion exchange HPLC for 24 h. The viability of Caco-2BBe cells was determined using the cell counting kit-8 (CCK-8) according to the manufacturer’s instructions. The cell viability was presented as the percentage of absorbance at 450 nm relative to the control group.

**Treatment of Caco-2bbe Cells and Evaluation of Anti-Inflammatory Activity of JBPH**

Caco-2BBe cells were seeded in a 48-well plate (0.2 × 10^4^ cells cm^-2^). TNF-α (40 ng/mL) was administered to the cells and incubated for 24 h. JBPH and the peptide fractions were added into the cell culture media 6 h prior to the TNF-α administration. Concentrations of JBPH and the peptide fractions used are described in figures. The IL-8 production in the cell culture media was determined by ELISA (Human CXCL8/IL-8 DuoSet ELISA; R&D systems) according to the manufacture’s instruction. To investigate the mechanisms underlying the JBPH-mediated anti-inflammatory effect, the cells incubated with and without TNF-α for 3 and 1 h were subjected to real-time reverse transcription-polymerase chain reaction (qRT-PCR) and immunoblot analyses, respectively, as described below.

**qRT-PCR Analysis**

Total RNA was isolated from Caco-2BBe cells using Sepasol^®^-RNA I Super G (Nacalai Tesque) and reverse-transcribed into cDNA using the ReverTra Ace qPCR RT kit (TOYOBO), in accordance with the manufacturers’ instructions. The PCR analysis was conducted using Thunderbird^®^ Next SYBR™ qPCR Mix (TOYOBO) in a StepOne Real-Time PCR system (Thermo Fisher Scientific). The primer sequences of *IL-8* and *GAPDH* used are provided in supplemental information (Table S1). The *IL-8* mRNA expression was quantified using the ΔΔC*t* method and normalized to glyceraldehyde-3-phosphate dehydrogenase (*GAPDH*) as the reference gene.

**Immunoblot Analysis**

Protein extracts of Caco-2BBe cells were prepared with the appropriate volume of lysis buffer [sodium dodecyl sulfate (SDS; 10 g/L), Triton X-100 (10 mL/L), and sodium deoxycholate (1 g/L) in 30 mmol/L Tris with protease and phosphatase inhibitors at pH 7.4] and mixed with half a volume of Laemmli sample buffer (3× concentrated), and heated to 95 °C for 10 min [3]. Protein extracts (20 µg) were separated by SDS-polyacrylamide gel electrophoresis and transferred to polyvinylidene difluoride membranes (Immobilon^®^-P, Merck Millipore). The membranes were blocked with 50 mg/mL skim milk for 1 h and incubated with primary antibodies at 4 °C for 16 h, followed by incubation with appropriate secondary antibodies for 1 h. Blots were developed using enhanced chemiluminescence detection reagent (Western-Lightning Plus-ECL, PerkinElmer, Waltham, MA, USA) in the Amersham Imager 680 (Cytiva, Uppsala, Sweden). Signal intensity of specific bands was quantified using ImageJ (National Institutes of Health, Bethesda, MD, USA). Total protein levels were visualized by Ponceau S staining to normalize the protein expression [4].

**Statistical analysis**

Data are presented as mean±SEM. Excel, version 16.67 (Microsoft Corp., Redmond, WA, USA), along with the Real Statistics Resources Pack (version 8.5) add-ins (https://real-statistics.com), were used for statistical analyses. One-way analysis of variance (ANOVA) was used to determine statistical significance, followed by Dunnett's or Tukey-Kramer's post-hoc test. *P* values less than 0.05 were considered statistically significant.

**Table S1.** Primer sequences for qRT-PCR

| Target genes | Forward (5ʹ to 3ʹ) | Reverse (5ʹ to 3ʹ) |
| --- | --- | --- |
| Human *IL-8* | TCTCAGCCCTCTTCAAAAACTTCTC | ATGACTTCCAAGCTGGCCGTGGCT |
| Human *GAPDH* | CAACGGATTTGGTCGTATTGGG | AAG GGG TCA TTG ATG GCA AC |

**Table S2.** Identified peptide sequence from fraction P1 by *de novo* LC/MS

| No | Parent protein | Peptide | m/z | RT | Area | ALC (%) | Error (ppm) |
| --- | --- | --- | --- | --- | --- | --- | --- |
|  |  |  |  |  |  |  |  |
| 1 | Chain B, Concanavalin-A (YKETNTIL) | YKETNTLL | 491.2665 | 15.54 | 3.62E+08 | 87 | 0.7 |
| 2 | NF | VRVGL | 272.1852 | 14.93 | 2.88E+08 | 96 | 3.4 |
| 3 | NF | FKKDPL | 374.224 | 13.75 | 1.48E+08 | 97 | 1.2 |
| 4 | NF | FNRYGSL | 428.7191 | 15.34 | 1.34E+08 | 98 | -0.2 |
| 5 | NF | HLLYNSVDKRLSA | 505.9492 | 14.49 | 1.18E+08 | 90 | 3.4 |
| 6 | NF | KTNDTPVTGHLKDF | 524.9399 | 14.97 | 1.14E+08 | 94 | 2.2 |
| 7 | Canavalin (KMPKDQIQEI) | KMPKDQLQEL | 615.3339 | 14.84 | 1.10E+08 | 88 | 3.9 |
| 8 | NF | VPEF | 491.2488 | 16.5 | 1.07E+08 | 92 | -2.4 |
| 9 | NF | YKRTLPTPVPV | 635.8806 | 15.63 | 1.05E+08 | 93 | 1.8 |
| 10 | NF | HLLY | 545.3099 | 14.69 | 1.01E+08 | 85 | 3.2 |
| 11 | Canavalin (KLDQGDAIKI) | KLDQGDALKL | 550.8193 | 15.3 | 9.75E+07 | 85 | 0.3 |
| 12 | NF | PVAL | 399.2603 | 15.73 | 9.46E+07 | 93 | 0.4 |
| 13 | Chain B, Concanavalin-A (PSYPHIGID) | PSYPHLGLD | 499.7513 | 16.31 | 8.48E+07 | 97 | 1.1 |
| 14 | Canavalin (KENVIRQIPR) | KENVLRQLPR | 418.2542 | 13.61 | 7.50E+07 | 89 | -0.3 |
| 15 | NF | MGGLL | 490.2693 | 17.44 | 6.67E+07 | 89 | 0 |
| 16 | NF | YRVLE | 340.1931 | 14.55 | 6.67E+07 | 96 | 2.2 |
| 17 | NF | LRKPEL | 378.2428 | 13.8 | 6.11E+07 | 96 | 1.2 |
| 18 | NF | VMDRPDVV | 465.7401 | 14.97 | 5.81E+07 | 86 | 1.7 |
| 19 | NF | LKLQAGTPF | 487.7857 | 16.45 | 5.54E+07 | 95 | -2.8 |
| 20 | NF | NLANPSRADF | 552.7759 | 15.39 | 5.25E+07 | 96 | 1.2 |
| 21 | Canavalin | FKNQHGSLRL | 600.3373 | 14.07 | 5.23E+07 | 94 | 2.5 |
| 22 | NF | VKYNGNWGPL | 574.2992 | 17.1 | 5.11E+07 | 96 | 1.4 |
| 23 | NF | PLLLGAHE | 424.7535 | 15.16 | 5.02E+07 | 95 | 1.1 |
| 24 | Canavalin | FSKNFL | 378.2071 | 16.4 | 4.98E+07 | 92 | -2.3 |
| 25 | NF | NSVHLPAL | 425.7426 | 16.7 | 4.81E+07 | 95 | -0.2 |
| 26 | Canavalin | SAFSKNFL | 457.2422 | 16.9 | 4.63E+07 | 85 | -0.7 |
| 27 | NF | VPEFL | 604.3341 | 18.65 | 4.31E+07 | 95 | 0.1 |
| 28 | NF | NTPVRKLEKL | 599.3696 | 13.85 | 4.08E+07 | 91 | 0.5 |
| 29 | Canavalin | LSSTKRLPSYL | 632.8679 | 15.73 | 3.60E+07 | 96 | 1.9 |
| 30 | NF | LLLPH | 296.6952 | 15.83 | 3.48E+07 | 93 | 2.3 |
| 31 | NF | YGRLDV | 361.6964 | 15.07 | 3.33E+07 | 92 | 3.2 |
| 32 | NF | YKLV | 261.669 | 14.64 | 3.32E+07 | 90 | 4 |
| 33 | Canavalin | FLSSTKRLPS | 568.3276 | 14.22 | 3.23E+07 | 98 | 0.9 |
| 34 | NF | NSVHLPA | 369.2021 | 14.36 | 3.13E+07 | 89 | 3.8 |
| 35 | NF | YGRLDLL | 425.2451 | 17.05 | 3.09E+07 | 89 | 0 |
| 36 | NF | RLSGDEL | 395.2101 | 14.45 | 2.66E+07 | 86 | 3.5 |
| 37 | NF | RLFG | 492.2942 | 14.6 | 2.65E+07 | 85 | 2.7 |
| 38 | NF | PAVPHL | 317.1901 | 15.59 | 2.57E+07 | 86 | 1.6 |
| 39 | NF | DLKLL | 301.1994 | 16.79 | 2.53E+07 | 88 | -0.7 |
| 40 | Canavalin | FLSSTKRLPSYL | 706.4004 | 16.45 | 2.44E+07 | 98 | -0.6 |
| 41 | NF | FLSNLD | 708.3569 | 16.16 | 2.24E+07 | 92 | 0.9 |
| 42 | NF | LKDF | 261.6507 | 14.4 | 2.18E+07 | 92 | 3.8 |
| 43 | NF | LRLA | 236.6666 | 14.31 | 2.17E+07 | 94 | 3.6 |
| 44 | Chain A, Concanavalin B | PYLPDL | 717.3842 | 18.86 | 2.17E+07 | 93 | 3.5 |
| 45 | Chain B, Concanavalin-A (NQFSKDQKDLI) | NQFSKDQKDLL | 668.3495 | 15.2 | 2.06E+07 | 87 | 1 |
| 46 | NF | HKFTHL | 261.4818 | 13.18 | 2.02E+07 | 86 | 0 |
| 47 | Canavalin (PIYSNNYGKL) | PLYSNNYGKL | 584.8042 | 15.88 | 1.95E+07 | 95 | 1.3 |
| 48 | Chain B, Concanavalin-A | SKDQKDLL | 473.7636 | 13.94 | 1.92E+07 | 96 | -0.4 |
| 49 | NF | YPLDL | 619.3467 | 18.21 | 1.86E+07 | 86 | 2.7 |
| 50 | NF | DGLHF | 294.6414 | 16.41 | 1.81E+07 | 96 | -3.6 |
| 51 | NF | LYSPF | 626.3201 | 18.21 | 1.66E+07 | 89 | 2.6 |
| 52 | NF | LPVPSL | 625.3942 | 18.16 | 1.47E+07 | 93 | 3.7 |
| 53 | NF | KVDKLFNRKEE | 468.9359 | 13.01 | 1.42E+07 | 94 | -1.9 |
| 54 | Canavalin | SAFSKNF | 400.7007 | 15.54 | 1.42E+07 | 92 | 0.5 |
| 55 | NF | TKVEL | 295.1824 | 14.45 | 1.38E+07 | 93 | 3.5 |
| 56 | NF | FGLM | 467.2322 | 18.38 | 1.30E+07 | 86 | 0 |
| 57 | Canavalin | KNQHGSLRL | 526.8013 | 13.43 | 1.09E+07 | 90 | -0.5 |
| 58 | NF | KKLFA | 303.7028 | 13.66 | 1.06E+07 | 90 | 1.6 |
| 59 | NF | LQMNEQLFVHP | 678.3451 | 17.8 | 1.05E+07 | 94 | 3.9 |
| 60 | NF | KFTHL | 323.1893 | 13.75 | 1.03E+07 | 89 | -0.7 |
| 61 | NF | LTGLF | 550.3235 | 18.54 | 9.51E+06 | 92 | 0 |
| 62 | NF | FRSNKF | 266.8131 | 13.48 | 9.07E+06 | 95 | -1.2 |
| 63 | Chain B, Concanavalin A | LGLFPD | 661.3561 | 19.53 | 8.61E+06 | 90 | 0.9 |
| 64 | NF | LAVGGF | 563.3199 | 17.59 | 8.57E+06 | 98 | 2 |
| 65 | NF | FKKDLP | 747.4403 | 13.75 | 8.42E+06 | 95 | 0.6 |
| 66 | Canavalin | SKNFLEA | 808.4213 | 15.7 | 8.29E+06 | 87 | 1.7 |
| 67 | NF | PVKLP | 277.1897 | 15.02 | 8.00E+06 | 96 | 2.3 |
| 68 | NF | FRKHLLA | 884.5458 | 13.38 | 7.83E+06 | 89 | -0.8 |
| 69 | NF | YNSHATKLAV | 552.2964 | 13.9 | 7.82E+06 | 95 | 1 |
| 70 | NF | LLNPDNNQNLRLL | 768.9291 | 17.25 | 7.53E+06 | 86 | 1.1 |
| 71 | NF | WTGF | 510.236 | 18 | 7.38E+06 | 95 | 2.6 |
| 72 | NF | LELL | 487.3145 | 18 | 7.01E+06 | 92 | 3.8 |
| 73 | NF | YTLF | 543.2787 | 18.59 | 5.45E+06 | 93 | -4.9 |
| 74 | NF | LEVNPYL | 847.4583 | 18.05 | 3.35E+06 | 95 | 2.7 |
| 75 | Chain B, Concanavalin-A | SWSFTSKL | 478.2484 | 17.68 | 1.56E+06 | 92 | 1.3 |
| 76 | NF | PKRELLKE | 337.8814 | 12.41 | 7.39E+05 | 96 | -1.1 |
| 77 | Canavalin | FKNQHGSLR | 362.8651 | 12.48 | 8.26E+04 | 87 | 0.5 |

NF, not found; RT, retention time; ALC, average local confidence

**Table S3.** Identified peptide sequence from fraction P2 by *de novo* LC/MS

| No | Parent protein | Peptide | m/z | RT | Area | ALC (%) | Error (ppm) |
| --- | --- | --- | --- | --- | --- | --- | --- |
|  |  |  |  |  |  |  |  |
| 1 | NF | DVDL | 461.2238 | 16.18 | 4.91E+08 | 95 | -0.8 |
| 2 | Canavalin | LTFPGSGEE | 936.4327 | 16.89 | 3.61E+07 | 95 | 2 |
| 3 | NF | HLWE | 584.2828 | 14.83 | 2.30E+07 | 97 | 0.2 |
| 4 | NF | LAFPGSGEE | 906.4213 | 16.75 | 1.81E+07 | 95 | 1.1 |
| 5 | NF | LESEF | 624.2876 | 16.13 | 1.37E+07 | 96 | 0.2 |
| 6 | NF | HFDY | 581.2361 | 14.88 | 1.03E+07 | 96 | 1.1 |
| 7 | Chain B, Concanavalin-A | YAPVHLWE | 507.7556 | 17.12 | 9.20E+06 | 98 | -0.3 |
| 8 | NF | TLFPGSGEE | 468.7194 | 16.89 | 9.06E+06 | 92 | 0.8 |
| 9 | NF | FVDF | 527.2499 | 18.11 | 6.63E+06 | 96 | -0.1 |
| 10 | NF | NWDLN | 661.2944 | 17.72 | 6.20E+06 | 90 | 0.6 |
| 11 | NF | YAPVHLMAE | 515.7534 | 16.61 | 6.04E+06 | 87 | -3.1 |
| 12 | NF | WGLE | 504.2451 | 17.64 | 5.94E+06 | 97 | -0.2 |
| 13 | Chain B, Concanavalin-A | YAPVH | 293.6531 | 12.8 | 5.00E+06 | 96 | 1 |
| 14 | NF | DPFSLD | 693.3092 | 18.04 | 4.30E+06 | 97 | 0.3 |
| 15 | NF | YTDF | 545.2239 | 16.08 | 3.82E+06 | 97 | -0.6 |
| 16 | Chain B, Concanavalin-A (LGLFPDAN) | LGLFPDAD | 847.4196 | 19.27 | 3.19E+06 | 95 | 0.1 |
| 17 | NF | FDYL | 557.2605 | 17.99 | 2.83E+06 | 94 | -0.1 |
| 18 | NF | VSQEAGREGFEY | 686.313 | 15.52 | 2.72E+06 | 96 | 1 |
| 19 | NF | ETHNPFE | 437.1905 | 15.04 | 2.26E+06 | 94 | 0.1 |
| 20 | Canavalin | NEGRAEVEL | 508.754 | 15.2 | 2.16E+06 | 90 | 0 |
| 21 | NF | WALE | 518.2612 | 17.25 | 1.95E+06 | 98 | 0.6 |
| 22 | Chain B, α-mannosidase | DDYFPYADGRNA | 702.297 | 16.81 | 1.72E+06 | 87 | 0.4 |
| 23 | NF | LTLEE | 604.3193 | 15.66 | 1.72E+06 | 97 | 0.8 |
| 24 | NF | VVEDF | 608.2927 | 16.32 | 1.55E+06 | 98 | 0.1 |
| 25 | NF | VEEF | 523.2397 | 15.89 | 1.53E+06 | 97 | -0.1 |
| 26 | NF | WTLE | 548.2714 | 17.18 | 1.33E+06 | 88 | -0.2 |
| 27 | Chain A, Concanavalin B | YWGQREDGL | 562.2622 | 16.46 | 1.21E+06 | 92 | 0.5 |
| 28 | NF | LVDDL | 574.3085 | 16.32 | 1.16E+06 | 97 | 0.4 |
| 29 | NF | VESEF | 610.2721 | 15.66 | 9.77E+05 | 95 | 0.4 |
| 30 | NF | TLLDTE | 691.3513 | 15.45 | 6.92E+05 | 96 | 0.7 |
| 31 | NF | VLSDHDF | 416.6957 | 15.45 | 5.42E+05 | 95 | 0.7 |

NF, not found; RT, retention time; ALC, average local confidence

**Table S4.** Identified peptide sequence from fraction P3 by *de novo* LC/MS

| No | Parent protein | Peptide | m/z | RT | Area | ALC (%) | Error (ppm) |
| --- | --- | --- | --- | --- | --- | --- | --- |
|  |  |  |  |  |  |  |  |
| 1 | Chain B, Concanavalin A | DVDLDNV | 395.1846 | 16.89 | 1.69E+07 | 95 | -0.6 |
| 2 | NF | ANPRHLDAGGKA | 402.8829 | 12.02 | 1.31E+07 | 94 | 0.4 |
| 3 | NF | DVDF | 495.2081 | 16.84 | 1.19E+07 | 98 | -0.8 |
| 4 | NF | FEEF | 571.2404 | 17.25 | 7.34E+06 | 98 | 0.9 |
| 5 | NF | FEEL | 537.2553 | 16.7 | 5.14E+06 | 98 | -0.4 |
| 6 | NF | VYDNEL | 752.3474 | 15.82 | 4.74E+06 | 97 | 1.8 |
| 7 | NF | FDLDA | 580.2612 | 16.7 | 3.48E+06 | 92 | -0.1 |
| 8 | NF | FADGEL | 651.2985 | 16.51 | 3.35E+06 | 90 | 0.2 |
| 9 | NF | TVEDF | 610.2721 | 15.96 | 2.29E+06 | 97 | 0.4 |
| 10 | NF | ANRPHLDAGGKA | 603.8204 | 12.02 | 2.01E+06 | 88 | -0.1 |
| 11 | NF | ADPRHLDAGGKA | 403.2107 | 12.17 | 1.84E+06 | 96 | -0.3 |
| 12 | NF | EEFTGF | 729.3093 | 18.1 | 1.54E+06 | 94 | 0.5 |
| 13 | NF | HLWE | 584.2831 | 14.79 | 1.36E+06 | 97 | 0.6 |
| 14 | NF | NAPRHLDAGGKAR | 454.9166 | 11.66 | 1.10E+06 | 93 | 0.4 |
| 15 | NF | YEGLDF | 743.3251 | 18.48 | 7.88E+05 | 88 | 0.7 |
| 16 | NF | FRKHLLA | 442.7772 | 13.38 | 7.69E+05 | 90 | 0.8 |
| 17 | NF | DAPRHLD | 412.2067 | 12.09 | 5.74E+05 | 96 | 0.6 |
| 18 | NF | KSNFLEASYD | 587.275 | 16.43 | 4.05E+05 | 86 | 0.5 |
| 19 | NF | DLDF | 509.2238 | 18.44 | 2.06E+05 | 89 | -0.7 |

NF, not found; RT, retention time; ALC, average local confidence

**Table S5.** Predicted probability score from PreTP-EL

| No | Parent protein | Peptide | Predicted probability score |
| --- | --- | --- | --- |
| 1 | NF | LRKPEL | 0.4849 |
| 2 | NF | PLLLGAHE | 0.4120 |
| 3 | NF | NTPVRKLEKL | 0.4770 |
| 4 | NF | LLLPH | 0.4463 |
| 5 | Canavalin | FLSSTKRLPSYL | 0.4826 |
| 6 | NF | FRKHLLA | 0.4875 |
| 7 | NF | LLNPDNNQNLRLL | 0.4956 |
| 8 | NF | LELL | 0.4352 |

NF, not found


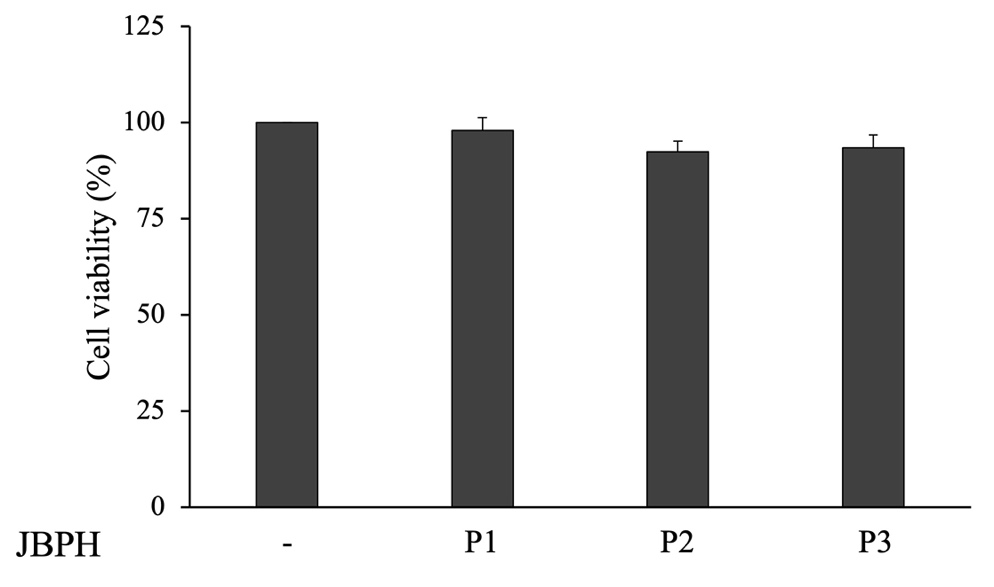


**Fig. S1** Effect of JBPH on viability of Caco-2BBe cells. Cells were incubated with JBPH for 24 h and the cell viability was evaluated using a commercially available kit. Values are presented as the mean ± standard error of mean (n = 6).

**References**

1. Wijatniko BD, Murdiati A (2019) Antioxidant activity of bioactive peptides derived from the hydrolysates of jack bean (Canavalia ensiformis (L.) DC.) protein isolate. AIP Conf. Proc. 2099, 020028. https://doi.org/10.1063/1.5098433

2. Rini DM, Yamamoto Y, Suzuki T (2023) Partially hydrolyzed guar gum upregulates heat shock protein 27 in intestinal Caco-2 cells and mouse intestine via mTOR and ERK signaling. J Sci Food Agric 103(10):5165–5170

3. Oyama M, Van Hung T, Yoda K, He F, Suzuki T (2017) A novel whey tetrapeptide IPAV reduces interleukin-8 production induced by TNF-α in human intestinal Caco-2 cells. J Funct Foods 35:376–383

4. Gilda JE, Gomes A V (2013) Stain-Free total protein staining is a superior loading control to b-actin for Western blots. Anal Biochem 440(2):186–188
